# Supplementary material for: MetaRibo-Seq measures translation in microbiomes
Source: Nat Commun. 2020 Jun 29;11:3268. doi: 10.1038/s41467-020-17081-z (PMC7324362; doi:10.1038/s41467-020-17081-z)
Supplement: Supplementary file 10 — Supplementary Data 7 [file 41467_2020_17081_MOESM10_ESM.zip › File2/Confidence_High_Taxonomy/293015_out.krona.html]

Javascript must be enabled to view this page.

members
magnitude
magnitudeUnassigned
count
unassigned
taxon
rank

293015\_out

20

20
superkingdom
2

20

SRS1054691\_contig\_number\_6269SRS893383\_contig\_number\_23557
1239
phylum
2

18
class
186801

18
186802
order

31979
family
17

17
genus
1485

species
59620

SRS014683\_contig\_number\_contig-100\_10117.58346SRS015065\_contig\_number\_contig-100\_7309.153145SRS015854\_contig\_number\_contig-100\_2559.2559SRS015890\_contig\_number\_22174SRS019397\_contig\_number\_contig-100\_782.36531SRS043411\_contig\_number\_contig-100\_2021.48285SRS049712\_contig\_number\_14826SRS063040\_contig\_number\_5356SRS077194\_contig\_number\_23235SRS077730\_contig\_number\_17305SRS097889\_contig\_number\_6449SRS098073\_contig\_number\_3670SRS104636\_contig\_number\_contig-100\_24885.24886SRS146812\_contig\_number\_40618SRS147346\_contig\_number\_61075SRS147766\_contig\_number\_17512SRS148874\_contig\_number\_355
17

1
family
186803


SRS143181\_contig\_number\_695
1
species
39491
